# Out-of-pocket expenditures and care time for children with Down Syndrome: A single-hospital study in Mexico City

**DOI:** 10.1371/journal.pone.0208076

**Published:** 2019-01-10

**Authors:** Silvia Martínez-Valverde, Guillermo Salinas-Escudero, Constanza García-Delgado, Juan Garduño-Espinosa, Verónica F. Morán-Barroso, Víctor Granados-García, Ma. Teresa Tiro-Sánchez, Filiberto Toledano-Toledano, Ma. Vanessa Aldaz-Rodríguez

**Affiliations:** 1 Centro de Estudios Económicos y Sociales en Salud Hospital Infantil de México Federico Gómez, Instituto Nacional de Salud, Mexico City, Mexico; 2 Departamento de Genética Hospital Infantil de México Federico Gómez Instituto Nacional de Salud, Mexico City, Mexico; 3 Dirección de Investigación Hospital Infantil de México Federico Gómez Instituto Nacional de Salud, Mexico City, Mexico; 4 Unidad de Investigación Epidemiológica y en Servicios de Salud Área Envejecimiento Instituto Mexicano del Seguro Social, Mexico City, Mexico; 5 Servicio de Urgencias Hospital General de Zona No. 24 Instituto Mexicano del Seguro Social, Mexico City, Mexico; 6 Unidad de Investigación en Medicina Basada en Evidencias Hospital Infantil de México Federico Gómez Instituto Nacional de Salud, Mexico City, Mexico; 7 Programa de doctorado en Administración y Sistemas de Salud de la Universidad Nacional Autónoma de México, Mexico City, Mexico; University of Nebraska Medical Center, UNITED STATES

## Abstract

**Aim:**

To examine the burden of out-of-pocket household expenditures and time spent on care by families responsible for children with Down Syndrome (DS).

**Methods:**

A cross-sectional analysis was performed after surveying families of children with DS. The children all received medical care at the Hospital Infantil de México Federico Gomez (HIMFG), a National Institute of Health. Data were collected on out-of-pocket household expenditures for the medical care of these children. The percentage of such expenditure was calculated in relation to available household expenditure (after subtracting the cost of food/housing), and the percentage of households with catastrophic expenditure. Finally, the time spent on the care of the child was assessed.

**Results:**

The socioeconomic analysis showed that 67% of the households with children with DS who received medical care in the HIMFG were within the lower four deciles (I-IV) of expenses, indicating a limited ability to pay for medical services. Yearly out-of-pocket expenditures for a child with DS represented 27% of the available household expenditure, which is equivalent to $464 for the United States dollars (USD). On average, 33% of families with DS children had catastrophic expenses, and 46% of the families had to borrow money to pay for medical expenses. The percentage of catastrophic expenditure was greater for a household with children aged five or older compared with households with younger children. The regression analysis revealed that the age of the child is the most significant factor determining the time spent on care.

**Conclusions:**

Some Mexican families of children with DS incur substantial out-of-pocket expenditures, which constitute an economic burden for families of children who received medical care at the HIMFG.

## Introduction

Down syndrome (DS), caused by the trisomy of the critical region of chromosome 21 in 21q22 [1], represents the main source of intellectual disability with a genetic origin [2, 3]. The estimated prevalence of DS is 1/792 newborns in the United States (US) [4]. In Mexico, estimates for 2007 ranged from 1/650 to 1/879 newborns [5]. One year later, it had declined in the country as a whole to 3.73/10,000 births [6], and in Mexico City it had fallen to 4.63 or 1/2,167 in the same period.

Patients with DS require personalized follow-up and health care services. They present multiple comorbidities including congenital heart disease and other gastrointestinal disorders, often requiring surgery during the first few years of life that can result in medical costs [7]. As a result of the variety of possible medical conditions, children with DS comorbidities such as congenital heart disease require specialized medical attention, with these children requiring at least three times as many medical appointments and hospitalizations than children without DS [8–10]. In addition, children with DS require non-medical services and support associated with their emotional and psychomotor development [11, 12].

These requirements may generate a greater need for health services and therefore more expenses for their respective families. An additional burden for families of children with disabilities is the considerable investment of time required to care for them, which can affect their available economic resources. The income of affected families is often insufficient to pay for medical care in the medium and long run [13]. For example, a survey report from the United States (US) estimated that 52% of a sample of 78,771 families caring for children with disabilities had difficulty paying medical bills compared with only 32% of families with children without disabilities [14]. According to another analysis, up to 40% of families having children with special health needs experienced a financial burden related to medical care [15]. In another study about DS, the mean total out-of-pocket annual cost was found to be greater among families of children with DS compared to those whose children did not have DS [16].

Health care services for Mexican families frequently implies out-of-pocket expenses [17].Thus, providing health care services and caring for children with more complex medical conditions can leave households vulnerable to elevated medical care expenses that often reach catastrophic proportions [18, 19]. The Mexican health care system is composed of public and private institutions that serve the total population of approximately 120 million people. Private institutions offer health care to people with the ability to pay for it. Minors who are insured by private health plans comprise only 2.7% of the population (about 1.11 million) [20, 21].

Heath care financed with public funds is two-tiered. Firstly, “social security” institutions afford health care, required by law as a benefit furnished by all large employers, for both private and government workers. Insurance benefits include: work-related disability pensions, paid maternity leave, and retirement pensions. The other type of publicly funded health care is provided by the Social Health Protection System (SPSS for its initials in Spanish) insurance program. It is an insurance open to the population not covered by Social Security schemes. This includes the self-employed, workers in the informal sector, the unemployed, and individuals who do not work. Through a network of public hospitals and clinics, this institution covers about 59.65 million people, including 23.09 million minors (0–18 years of age) [20, 21].

The SPSS health care program is an insurance plan limited in terms of benefits, offering a package of mainly primary and secondary health care services. The budget for the SPSS does not cover all the care required for chronic disorders like DS and birth defects, for example. For this reason, a presidential initiative, named the "Seguro Médico Siglo XXI" (SMS XXI) or “21^st^ Century Medical Insurance”, was launched to provide additional funding for this purpose. In conjunction with the *Fund for Protection against Catastrophic Health Expenditures* (FPGC for its initials in Spanish), SMS XXI covers high specialty (3rd level) medical interventions for families affiliated with the SPSS [20].

The SMS XXI, which is funded by the FPGC, affords extended neonatal intensive care, surgical procedures, and ambulatory 2^nd^ and 3^rd^ level attention to all children from birth to five years old, an affiliated population representing approximately 7.44 million children. However, children from the ages of 6 to 18 years (around 16.5 million children) are not beneficiaries of SMS XXI. As a result, their families have to make the required payments and co-payments for medical care in public hospitals. Additionally, these families must cover other out-of-pocket health-related expenses like medications and laboratory studies that are not included in the SPSS [22].

Unfortunately, to our knowledge, no studies have been performed in Mexico specifically regarding out-of-pocket expenditures per family for children with neuro-diverse development. Consequently, estimates of the resources that would be necessary for families providing care for a child with DS are uncertain. This uncertainty makes it difficult to identify the means to improve the standard of living for the family and child living with DS.

A substantial segment of the population is vulnerable to the economic burden resulting from the provision of health care to children with DS or other disabilities. There is evidence suggesting that the extra care required either drives many families to impoverishment due to the magnitude of out-of-pocket expenditures [23] or diminishes the quality of life of families already on the lowest rungs of the socioeconomic ladder. The effects of poverty are associated not only with reduced income but also emotional stress. All these factors accumulate over time and generate complex economic conditions in the affected households [24].

The Hospital Infantil de México Federico Gomez (HIMFG) is one of the National Institutes of Health that receives patient referrals from all states of the country and provides medical care to children without access to social security medical services. Consequently, it provides medical care to families on the lowest rung of the socioeconomic ladder. In addition to providing patient care, it is a research and teaching center. The aim of the present study, carried out in the aforementioned institution, was to estimate the out-of-pocket household expenditures dedicated to the medical care of children with DS. These expenses were classified by the age of the child and the decile of *per capita* expenditure. Additionally, the percentage of households with catastrophic expenditures and the time spent caring for these children were ascertained.

## Materials and methods

### Study design and subjects

A cross-sectional economic study was conducted to examine the out-of-pocket household expenditures related to medical care for children with DS. The amount of the expenditure was categorized according to the socioeconomic status of the families (using standard criteria for Mexico) and the age of the child in question. Moreover, calculations were made for the percentage of households with catastrophic expenditures and the time spent caring for the child. The study was approved by the Research, Ethics and Biosafety Committee of the HIMFG (register number HIM/2009/004).

A convenience sample was used, and data were collected in 2009. The study involved 50 families of children with DS diagnosed in the outpatient clinic (department of genetics) at the HIMFG National Institute of Health in Mexico City. From 2007 to 2009, this institution received approximately 100 new outpatient cases per year of children with DS under 18 years of age. The sample used for this study consisted of families whose children had DS and received medical care at the hospital for at least one year. The inclusion criteria for the participants (parents) were: being over 18 years old; direct kinship to a registered patient (a minor) with DS; the willingness to participate in the study; and completing the survey and signing the informed consent form. The survey collected data on household income and total expenditures, transportation and medical care-related expenses for the children with DS, and hours spent on caring for these children.

### Definitions

The variables comprise the total one-year out-of-pocket expenses related to the medical care of a child with DS, the possible catastrophic level of these expenses, the available household expenditure, the socioeconomic classification of households by deciles of *per capita* expenditure, and the time spent on care.

Total out-of-pocket expenditures of the household (*t-opexp-h*) include those linked to medical care and transportation for one year.

Out-of-pocket medical care expenditures *(opmcexp*) was defined as direct disbursements for the medical needs of the child with DS during the appointment at the HIMFG, such as medications, outpatient visits, prostheses, lab tests, clinical analyses, and hospitalizations [23, 25].

Transportation expenses (*texp)* were defined as those used for public and private transportation involved in making their way to the facility in which the child received medical care.

The magnitude of the total out-of-pocket expenditures of a household was defined as its percentage related to available household expenditures for one year.

*Available household expenditures* was defined as total household expenditures (*ahexp*, the proxy for permanent income) minus the cost of food and housing [23, 25]:

ahexp=thexp−(foodexp+hoexp)

Where,

*ahexp* is the available household expenditures,

*thexp* is the total household expenditures,

*foodexp* is food expenditures, *hoexp* is housing expenditures (rent, gas and electricity).

Catastrophic expenditures (*ctexp*) occurs if *total out-of-pocket expenditures* on medical care and transportation were greater than or equal to 30% of the available household expenditures (*ahexp*). C*texp* = 1 if *t-opexp-h* ≥ 0.3 *ahexp*, or *ctexp* = 0 if *t-opexp-h* < 0.3 *ahexp* [26, 27].

This indicator allowed us to establish the percentage of households with catastrophic expenditures. A threshold of 30% was utilized as some official Health Ministry reports use this threshold.

The socioeconomic level was classified according to the decile of *per capita* expenditures (using total household expenditures as a proxy for income, because households tend to underreport income). The deciles of average annual *per capita* expenditures were based on the cut-off points from the 2008 National Household Income and Expenditure Survey (Encuesta Nacional de Ingreso y Gasto de los Hogares, or ENIGH), reported by the national census institute (Instituto Nacional de Estadística y Geografía, or INEGI) in Mexico [28]. Before categorizing the households, all expenditure values were annualized and updated using the national consumer price index [29] for December 2015 and converted to United States dollars (USD) at the respective exchange rate (1 USD = 17.34 Mexican pesos) [30].

Out-of-pocket medical care expenditures was categorized by the age of the child with DS, in order to examine the median expenditures for three categories: children 0 < 1, 1−5 and >5-years-old. Differences between age groups were compared by using the Kruskal-Wallis test.

### Time spent on care

A regression analysis was performed to explain the hours spent caring for children with DS. The variables were the main diagnosis and associated comorbidities found in the medical files of children in the sample (congenital heart anomaly, hernia, duodenal atresia, and hearing and throat problems), sex, age, and the educational level of the parents. The normality of the dependent variable was graphically verified by using a histogram and the Shapiro-Wilk test. Analyses were conducted on Stata version 13.1 (StataCorp, College Station, TX, USA).

Therefore, a linear regression model was proposed to determine the hours dedicated to caring for children with DS:

$$Hours=\beta_{0}+\beta_{1}X_{1}+\beta_{2}X_{2\ldots }\;\beta_{4}X_{4}+\varepsilon_{ij}$$

Where X_1_ = the age of the child (in years); X_2._ = the sex of the child (1 for males, 2 for females); X_3_ = DS and associated comorbidities. For the latter parameter, the categorical variable was diagnosis with DS (= 1), and DS accompanied by a congenital heart anomaly (= 2), a hernia or duodenal atresia (= 3), and hearing and throat problems (= 4). Finally, X_4_ represented the educational level (expressed in grades completed) of the parents.

## Results

A total of 45 mothers who cared for children with DS were interviewed. Eighty-two percent reported living in a location that required them to travel more than 2½ hours to attend a medical appointment at the hospital. The average age of the children was 4 years. Only about half of the children attended school (in most cases, a public school) (Table 1). All the patients had neurological complications linked to intellectual disability. The other complications included cardiovascular events (55%), ophthalmologic disorders (53%), musculoskeletal disorders (33%), diseases of the oral cavity (31%), gastrointestinal ailments (27%), and endocrinological diseases (24%) (data not shown).

**Table 1:** Characteristics of children with Down Syndrome and of their parents.

	Children (n = 45)
	Average (Standard deviation)
Age (years)	4 (4.12)
Place of origin (%)	
Local	18
Out of the metropolitan area	82
Average time of transfer (hours)	
Local	1.22 (1)
Out of the metropolitan area	2.39 (0.58)
Children who attend school (%)	47
Type of education (%)	
Private	21.7
Public	78.3
Main reason for appointment (%)	
Routine pediatric check-up^a^	57
Emergency	23
Hospitalization	20
Health plan for medical care provided (%)	
“Seguro Médico Siglo XXI” Insurance Program	53
No funding by any health care program	47
Head of household (%)	
Male	87
Female	13
Employment for the head of household (%)	
Part time	33
Permanent	62
Not reported	5

^a^ Genetics and cardiology.

### Use of resources

The primary reason for a hospital visit was a pediatric check-up (57% of cases), followed by emergencies (23%). The remaining 20% were related to hospitalizations. On average, the families paid for five specialty medical appointments in one year for their child with DS. Lab tests during the previous 12 months were carried out on 90% of the patients for the diagnosis of associated diseases. The most frequent lab tests were thyroid profiles (67%), blood analyses (29%), karyotyping (20%), urinalyses (16%), metabolic panels and coagulation times (8%). The number of tests ranged from one to seven per year.

#### Socioeconomic classification by decile

Sixty-seven percent of the households were in the bottom four deciles of expenditures in Mexico, indicating that the ability of the families to meet medical care expenditures was limited (Table 2). The magnitude of the average total out-of-pocket expenditures was 27% of total available household expenditures (equivalent to US$464): 21% for out-of-pocket medical care expenditures and 6% for transportation costs (Table 2).

**Table 2:** Average annual out-of-pocket medical and transportation expenditures (USD) and percentage of total available household expenditures, the percentage of households with catastrophic expenditures, and average hours of care provided in families having children with Down Syndrome, categorized by deciles of average per capita total household expenditures.

Deciles	Average *per capita* total household expenditures in Mexico ^a^ ^¥^ (USD)	% of households in each decile ^1/^ (n = 45)	Average available expenditures of households in each decile (USD)	Annual out-of-pocket medical care expenditures (USD)	Annual out-of-pocket expenditures for transportation (USD)	Total out-of-pocket expenditures ^b^ (USD)	% of out-of-pocket expenditures for medical care	% of total out-of-pocket expenditures in regard to available expenditures ^b^	% of households with catastrophic expenditures	Average hours of care/week
I	513	16	991	112	62	174	11	18	14	72.1
II	726	18	1766	409	102	511	28	36	38	74.2
III	918	13	1985	450	31	481	28	29	50	70
IV	1117	20	2259	400	109	509	25	29	44	62.3
V	1339	9	1333	328	86	414	26	34	50	52.5
VI	1617	7	1819	106	50	155	5	8	0	84
VII	2006	11	3273	588	196	785	22	27	20	46.2
VIII	2623	6	4432	387	307	694	10	17	33	28
**Total** ^c^			**2035**	**358**	**107**	**464**	**21**	**27**	**33**	**63**

^a^ All prices were adjusted annually by using the national consumer price index for December 2015 and converted to USD at the respective exchange rate.

^**¥**^ These values were the cut-off points of each decile of *per capita* national expenditures as reported in the ENIGH 2008 Survey published by INEGI in Mexico.

^1/^ Households of children with DS were ranked according to the annual *per capita* expenditure reported in the ENIGH 2008.

^b^ The value of each line corresponds to the average of the decile.

^c^ The total refers to the average of the sample.

**Fig 1:**
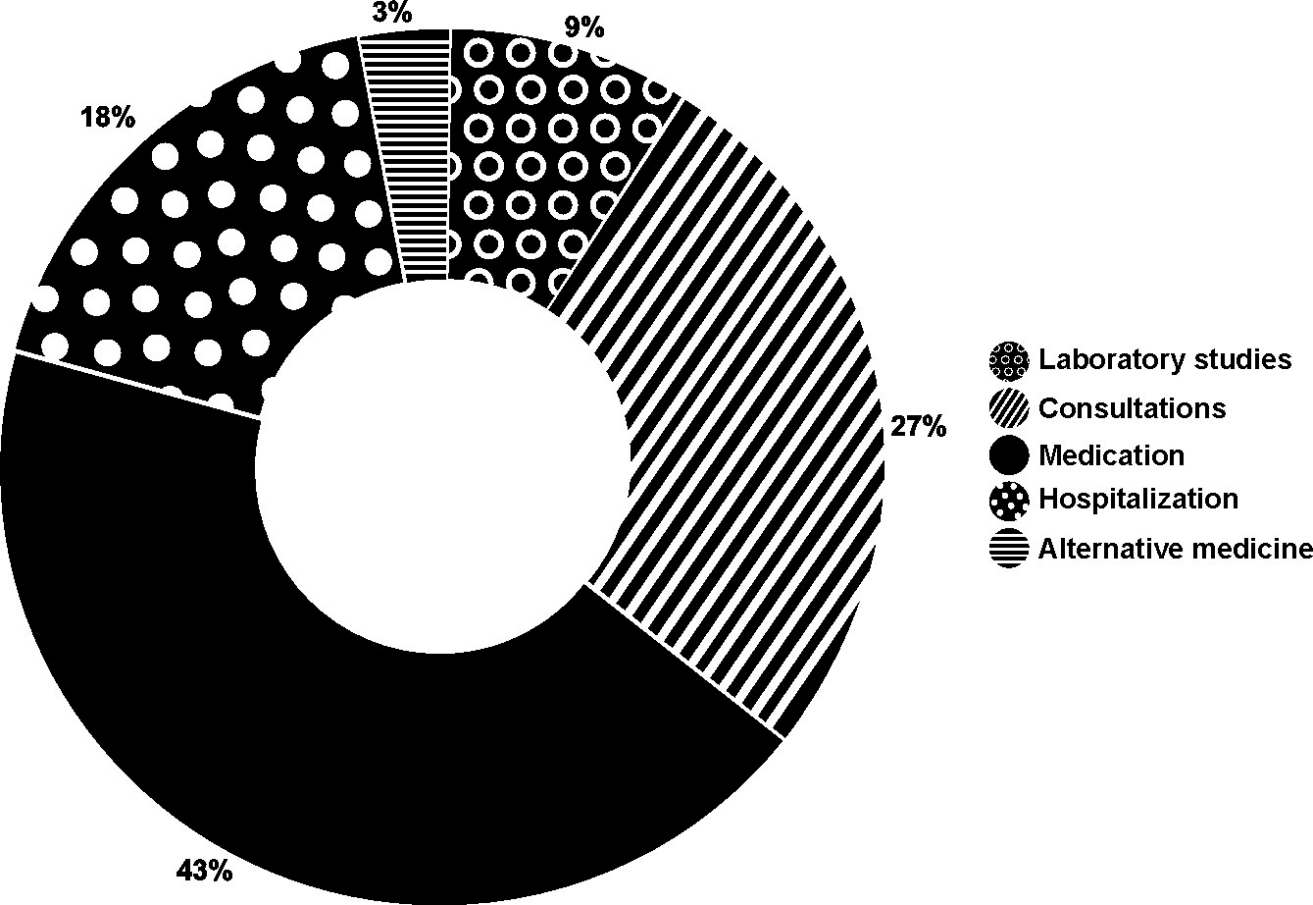
Main items of out-of-pocket expenditures in children with Down Syndrome. The largest share of out-of-pocket expenditures costs is for medication.

The items showing the highest proportion of expenditures (from the highest to the lowest) were medications, medical appointments, and hospitalizations. These expenses corresponded both to acute episodes and routine monitoring of the children (Fig 1).

### Health care expenditures by age category

Out-of-pocket medical care expenditures were substantial for all age groups. The median medical care expenditures was greater for households with children over the age of five (versus the other age groups; Table 3). However, when the groups were compared by using a median test (Kruskal-Wallis = 1.339, *p* = 0.5121), none of the differences were statistically significant.

**Table 3:** Median and interquartile range of annual out-of-pocket medical care expenditures (USD) by age category^a^.

Age group (years old)	Median	Interquartile range (IQR)	
0 <1	$96	62	75
1–5	$75	23	354
> 5	$162	69	572

^a^ All prices were adjusted annually by using the national consumer price index for December 2015 and converted to USD at the respective exchange rate. The chi-squared test indicated a lack of significant differences.

### Catastrophic expenditures

The odds ratio for catastrophic expenditures (1.22) was highest for households with children over versus under the age of five, but it was not statistically significant. Whereas 33% of the households incurred catastrophic expenditures, 46% reported the need for loans from relatives to cover medical care expenses (data not shown).

### Time spent on care

The regression analysis reveals that the average time of care per day was 11.22 hours, equivalent to 78.5 hours per week. The age of the child with DS best predicted the linear relationship of time spent on care. The older the child, the less time required for care (Table 4).

**Table 4:** Results of the regression analysis of time spent on care.

Dependent variables	Coefficients	*P > t*
Age of the child (number of years)	-0.4395	0.005
Gender (Male)	-0.9498	0.336
Associated comorbidities		
DS with congenital heart disease	2.497	0.105
DS with hernia or duodenal atresia	2.605	0.131
DS with hearing and throat problems	0.1493	0.950
Educational level of the parents	-0.2329	0.123
Constant	11.222	<0.001

DS = Down Syndrome.

The regression goodness of fit (R^2^) was 0.423, indicating that the variables in the model explain about 42% of the variability in the time spent on care; the age of the child is the most significant predictor. The results of the normality test were Shapiro-Wilk W = 0.9678, p-value = 0.24197. Thus, the null hypothesis of normality was not rejected. Heteroskedasticity was evaluated with the Breusch-Pagan/Cook-Weisberg test: Constant variance, Χ^2^ (1) = 0.63; prob > Χ^2^ = 0.4278. Hence, the null hypothesis of constant variance was not rejected. Multicollinearity was assessed with the mean of the variance inflation factor (VIF) = 1.95, which does not suggest multicollinearity was high.

## Discussion

The families in the current sample sought medical care services at the HIMFG for their child with DS. The total out-of-pocket expenditures for these children were examined. Data were reported for household socioeconomic status categorized by deciles, the percentage of families of children with DS who received medical care at HIMFG and who incurred catastrophic expenditures, the proportion that resorted to borrowing money from relatives to pay medical expenses, medical care expenditures by age category, and the time spent on the care of the child with DS. The results reveal that most of the families in our data had to cope with burdensome expenditures related to the medical care of their child with DS. To a large extent, this seems to stem from their socioeconomic status, because most of them are in the lower deciles of household expenditures. Although this is a convenience sample and it is not a representative population of all Mexican families having children with DS, total out-of-pocket expenditures constituted approximately one-quarter (between 21% and 27%) of available household expenditures. In addition, one-third of the households (33%) incurred catastrophic expenditures and 46% were forced to obtain loans from relatives to finance medical care (Table 2). These percentages represent a risk of impoverishment faced by the families of children with DS. The high proportion of out-of-pocket expenditures as a percentage of available household expenditures resulted from the continuous nature of spending on medical events and the low income of most families in our present sample. The consequence may be a substitution effect on the composition of household expenditures, meaning that families are forced to spend money on medical care bills that otherwise would be destined for education, clothing, cleaning and so forth [25, 31].

Another aspect is that households of children with DS with the lowest total annual household expenditures (decile I) reported a lower proportion of out-of-pocket expenditures for health care than the other expenditure deciles in our data. The magnitude of expenditures was three times greater for households in deciles II-IV than those in decile I. For the other four deciles (V-VIII), there was no clear trend in reduced expenditures as a percentage of available household expenditure. Interestingly, another study demonstrated that health care costs are similar for high- and low-income families having children with DS [32]. Our data suggest that, as a result of their impoverished condition, the families in the bottom decile of expenditures probably do not satisfy the medical care needs of their children with DS.

In our study, the percentage of catastrophic expenditures was greater for a household with children over five years old compared with households with younger children. However, the increase in the medical care expenditures categorized by age was not statistically significant. If we were to choose an indicator of the extent that households are affected by out-of-pocket expenditures, we would consider the percentage of households with catastrophic medical expenditures.

The likely reason for catastrophic expenditures in the older group of children is that the federal government program, SMS XXI, guarantees only some health services for these children. The SMS XXI provides all t medical care for children under the age of five, although in the case of families with children over the age of five, their parents must pay for additional chronic disorder-related rehabilitation and other medical services. This program aims to finance comprehensive health care for children not covered under social security [22]. It is not clear whether the services provided to the children over the age of five are comprehensive enough because their parents must pay for additional rehabilitation and other medical services.

To our knowledge, no estimate has yet been made of the capacity of the infrastructure in Mexico to offer health care to minors with DS. Despite this shortcoming in our knowledge, it is understood that most minors with DS have special health care needs and both age groups of children should be included in insurance and health care coverage. The limitation of SMS XXI coverage of children with disabilities during the first five years of life implies inadequate access to health care services and a resulting financial burden for many families having children with DS over the age of five.

We recommend extending comprehensive health care coverage to children with DS who are over five years old in order to guarantee full access to medical attention and improve the financial condition of the respective families. The present analysis strongly suggests that families at the lower end of the economic ladder, especially those in the bottom decile of household expenditures, are likely to have to sacrifice spending on other necessities to provide health care for their child with DS. They may be able to meet only a part of the medical care needs of this child and are therefore likely to incur debt and/or catastrophic medical expenditures. In the United States, children with DS are considered to have special health care needs. Due to the increased risk of adverse chronic physical, developmental, behavioral, or emotional health conditions, they require more health care services than other children [13, 33].

Our study results furnish relevant information regarding low-income Mexican families of children with DS who are attended to in the HIMFG and suggest that the out-of-pocket expenditures of these families constitute a significant economic burden. However, because of the lack of a control group (children without DS), it was not possible to estimate the increase in time and money spent on medical care and special needs associated with DS. In the US, a large survey was conducted to estimate the additional expenses incurred by families of children with DS compared to families of children without disabilities, resulting in a monthly increase of US$84 [16].

Certain critical differences between the cited survey and the present investigation must be highlighted. The expenses of Mexican families in the current sample were concentrated on medications, appointments, prostheses, lab tests, clinical analyses, hospitalizations, and transportation. The SMS XXI insurance program does not cover all goods and services necessary for children with DS; this program reimburses certain high cost services, but not all. Families have to pay for the medical care not covered free of charge by the public plan, and this results in a considerable economic burden. Families in our study had very limited income, making it impossible for them to purchase a private health insurance plan to cover all the health needs of a child with DS.

In the US, the insurance system is different. Families pay for some medical costs in addition to co-payment of expenditures. Out-of-pocket payments are primarily for inpatient and outpatient care, emergency room visits, home health agency costs, and outpatient pharmacy costs. In addition, one report from the US specifically identified the medical care-related costs involved in the first year of life of a child with DS [16]. The most significant expenses were linked to clinical conditions, including cardiac events, gastrointestinal complications, and cataracts. Across all age categories, the out-of-pocket expenses for outpatient visits were not meaningfully different across patients with DS of varying ages [16]. In our results, differences in out-of-pocket expenditures by age categories were not statistically significant. However, the families of children with DS who received medical care at HIMFG incurred out-of-pocket expenses during the first years of childhood.

The economic burden from healthcare expenses is expected to be lower for an American family than a Mexican family having a child with DS. This is because Mexican families have a lower ability to afford medical care due to their lower income, and because the Mexican public healthcare system provides insufficient support. The poorest families in our study (those in the bottom decile) reported lower out-of-pocket expenditures compared to families in deciles II-IV, and this finding is likely due to the under-consumption of medical care caused by their extremely limited income.

Concerning the time spent caring for children with DS, the families of our sample indicated that they dedicated an average of 11 hours/day, more than an average working day. On the one hand, there are several reports of slightly less time dedicated to care (7.14−8.57 hours) for children with multiple disabilities on average [34]. On the other hand, one study found a 30% increase in care time for children with chronic diseases [35]. The most important implication in our data on care time is the likely negative effect on family income in the long run. Similarly, a US health survey revealed that families of children with multiple chronic diseases incur substantial debt to cover medical expenses [14]. An additional financial hardship for Mexican families having a child with DS is the necessity of the mother to abandon her economic activity because of the time spent on care. The difference between the two countries’ governmental infrastructure and social support provided to families raising children with DS is substantial between Mexico and the US, especially because, in the US, the majority of children with DS attend school [36]. Our findings suggest that this is not the case in our sample of Mexican children with DS. Of the sample of children surveyed, approximately 50% were attending school. This situation represents a burden for Mexican families caring for children with DS.

The average time for providing special care to children with other conditions (chronic illness, temporal illness, and disability) is 10.1 hours/day [37]. It must be taken into account that the estimate of 78.5 h/week reported in our data includes all the time spent on caring for the children (the normal time for care plus the extra time due to DS). Because there was no control group in our study, the time spent caring for children with neurotypical development was not available. Consequently, we were unable to calculate the increase in time for the care for children with DS relative to other children.

Our study has some limitations, the first of which is that our results on out-of-pocket medical expenses are not generalizable to all families of children with DS. Our study used a convenience sample of children with DS that used health services at the HIMFG. Another limitation was the lack of a control group, so we could not identify differences in health care expenditures made by families of children with and without DS and other clinical conditions. An additional limitation is the small sample size in our data, to conduct the statistical analysis of expenditures by age group. Notwithstanding these limitations, this research provides partial evidence of the health care expenditures that families of children with DS make on an ongoing basis and of the time they invest in their care.

### Conclusions

The current data suggest that out-of-pocket expenditures constitute a sizeable burden for Mexican families of children with DS who received medical care in the HIMFG. Households at the lowest decile of annual household expenditures may not be able to provide for all the medical needs of their child with DS. Families of children over the age of five are faced with a substantial percentage of catastrophic expenditures for medical care services, probably because they are excluded from a governmental program that covers comprehensive medical care for children under the age of five.

## Supporting information

S1 Supporting File Out of pocket expenses of families with Down Syndrome children who attended Hospital Infantil de México Federico Gomez. It includes all variables and expenses made by families in the sample.
(XLSX)

S2 Supporting File Time spend on care by families with DS children attending Hospital Infantil de México Federico Gomez. It includes characteristics of parents and caregivers and time spent in caring children in the sample.
(DTA)
